# Optimal health and disease management using spatial uncertainty: a geographic characterization of emergent artemisinin-resistant *Plasmodium falciparum* distributions in Southeast Asia

**DOI:** 10.1186/s12942-016-0064-6

**Published:** 2016-10-24

**Authors:** Eric P. M. Grist, Jennifer A. Flegg, Georgina Humphreys, Ignacio Suay Mas, Tim J. C. Anderson, Elizabeth A. Ashley, Nicholas P. J. Day, Mehul Dhorda, Arjen M. Dondorp, M. Abul Faiz, Peter W. Gething, Tran T. Hien, Tin M. Hlaing, Mallika Imwong, Jean-Marie Kindermans, Richard J. Maude, Mayfong Mayxay, Marina McDew-White, Didier Menard, Shalini Nair, Francois Nosten, Paul N. Newton, Ric N. Price, Sasithon Pukrittayakamee, Shannon Takala-Harrison, Frank Smithuis, Nhien T. Nguyen, Kyaw M. Tun, Nicholas J. White, Benoit Witkowski, Charles J. Woodrow, Rick M. Fairhurst, Carol Hopkins Sibley, Philippe J. Guerin

**Affiliations:** 1 WorldWide Antimalarial Resistance Network (WWARN), Oxford, UK; 2 Nuffield Department of Medicine, Centre for Tropical Medicine and Global Health, University of Oxford, Oxford, OX3 7LJ UK; 3 School of Mathematical Sciences, Monash University, Melbourne, Australia; 4 Department of Genetics, Texas Biomedical Research Institute, San Antonio, TX USA; 5 Mahidol-Oxford Tropical Medicine Research Unit, Mahidol University, Bangkok, Thailand; 6 Laboratory of Malaria and Vector Research, National Institute of Allergy and Infectious Diseases, National Institutes of Health, Rockville, MD USA; 7 Dev Care Foundation and Malaria Research Group, Dhaka, Bangladesh; 8 Spatial Epidemiology and Ecology Group, Department of Zoology, University of Oxford, Oxford, UK; 9 Hospital for Tropical Disease, Oxford University Clinical Research Unit, Ho Chi Minh City, Vietnam; 10 Defence Services Medical Research Centre, Naypyitaw, Myanmar; 11 Médecins Sans Frontières, Brussels, Belgium; 12 Department of Molecular Tropical Medicine and Genetics, Faculty of Tropical Medicine, Mahidol University, Bangkok, Thailand; 13 Malaria Molecular Epidemiology Unit, Institute Pasteur in Cambodia, Phnom Penh, Cambodia; 14 Shoklo Malaria Research Unit, Mahidol-Oxford Tropical Medicine Research Unit, Faculty of Tropical Medicine, Mahidol University, Mae Sot, Thailand; 15 Microbiology Laboratory, Lao-Oxford-Mahosot Hospital-Wellcome Trust Research Unit (LOMWRU), Mahosot Hospital, Vientiane, Lao People’s Democratic Republic; 16 Institute for Global Health, University of Maryland School of Medicine, Baltimore, MD USA; 17 Menzies School of Health Research, Charles Darwin University, Darwin, Australia; 18 Faculty of Tropical Medicine, Mahidol University, Bangkok, Thailand; 19 Myanmar Oxford Clinical Research Unit, Yangon, Myanmar; 20 Department of Genome Sciences, University of Washington, Seattle, WA USA; 21 Harvard TH Chan School of Public Health, Harvard University, Boston, USA

**Keywords:** Surveillance, Drug resistance, Malaria, Artemisinin, Greater Mekong Subregion

## Abstract

**Background:**

Artemisinin-resistant *Plasmodium falciparum* malaria parasites are now present across much of mainland Southeast Asia, where ongoing surveys are measuring and mapping their spatial distribution. These efforts require substantial resources. Here we propose a generic ‘smart surveillance’ methodology to identify optimal candidate sites for future sampling and thus map the distribution of artemisinin resistance most efficiently.

**Methods:**

The approach uses the ‘uncertainty’ map generated iteratively by a geostatistical model to determine optimal locations for subsequent sampling.

**Results:**

The methodology is illustrated using recent data on the prevalence of the *K13*-propeller polymorphism (a genetic marker of artemisinin resistance) in the Greater Mekong Subregion.

**Conclusion:**

This methodology, which has broader application to geostatistical mapping in general, could improve the quality and efficiency of drug resistance mapping and thereby guide practical operations to eliminate malaria in affected areas.

**Electronic supplementary material:**

The online version of this article (doi:10.1186/s12942-016-0064-6) contains supplementary material, which is available to authorized users.

## Background

The success of artemisinin combination therapies (ACTs) in combatting *Plasmodium falciparum* malaria parasites over the last decade is being increasingly compromised by the emergence of artemisinin-resistant parasites in Southeast Asia [1–7]. There appears to be a crucible of antimalarial resistance in Western Cambodia, where parasites have repeatedly evolved resistance to widely used antimalarial drugs [1]. Resistance to artemisinin, the latest casualty, not only jeopardises the elimination of *P. falciparum* malaria in this region but also poses a threat to global malaria control should resistant parasites spread to India and then Africa, a path previously taken by resistance to older antimalarials [8, 9]. While ongoing studies are measuring the extent of artemisinin resistance in the Greater Mekong Subregion (GMS), these are relatively time-consuming, logistically challenging, and costly. It is therefore important to identify the most informative sites for future data collection to accurately characterise the distribution of resistance in both time and space.

The recent identification of *K13*-propeller polymorphism as a genetic marker of artemisinin-resistant *P. falciparum* has the potential to enable rapid detection and geographical mapping of artemisinin-resistant parasites in the GMS [10]. While parasites with a nonsynonymous single-nucleotide polymorphism (SNP) in the *K13*-propeller are suspected of being artemisinin-resistant, this has been confirmed for only some of the many mutations observed in Cambodia, Vietnam, Thailand, Myanmar, and Southern China. Slow parasite clearance rates following treatment with an artemisinin monotherapy or an ACT [10–13] and increased parasite survival rates in ex vivo and in vitro ring-stage survival assays (RSAs) [14, 15] have both been utilized for this purpose. However, further studies are needed to more efficiently follow geographical trends in the prevalence of *P. falciparum* parasites carrying these *K13*-propeller mutations and provide timely intelligence to guide decisions aimed at reducing the emergence or spread of artemisinin resistance.

Elimination of artemisinin-resistant parasites requires the identification of geographical areas threatened by resistance, locations seen as potential hot spots for new outbreaks of drug resistant malaria [5, 16]. Such geospatial information is crucial for efficient mobilization of appropriate resources to eliminate resistant parasites and thereby reduce the risk of their spread to other localities. Since the geographical patterns of both transmission and resistance mutation frequency are highly heterogeneous, country-level decisions are unlikely to lead to optimally tailored strategies and uses of limited resources. Local information on the efficacy of ACT partner drugs assures that the most appropriate combination can be selected in a given region. There is a paramount need for geospatial maps to convey to policy makers spatial information on the current status of key parameters and patterns of drug resistance. The objective of our study is to demonstrate that a strategic modelling methodology, referred to as ‘smart surveillance,’ can enhance data visualisation through spatial mapping and maximise the efficiency of further sampling to produce a cost effective map of antimalarial drug resistance.

## Methods

Geospatial modelling techniques using molecular markers have already been applied to estimate the prevalence of antimalarial drug resistance [5, 7]. These models can also be used to identify geographical areas where data are currently insufficient for policy makers to determine whether or not drug resistance is present. In this study, we transform the geospatial mapping approach into a ‘smart surveillance’ methodology by utilizing the geospatial maps generated to identify optimal locations for additional sampling.

Our focus on Southeast Asia is motivated by the urgent need to provide evidence for ongoing malaria control and elimination efforts in the face of expanding artemisinin resistance in this region. The longer-term goal is to apply the methodology to other malaria-endemic regions. In all cases, reducing the uncertainty of current estimates of the geographical prevalence of antimalarial drug resistance is balanced against limitations on available resources. There is a practical constraint on the number of new sites that can be sampled, and therefore a pragmatic requirement to ensure that sites are selected effectively and efficiently.

### Data sources

We have used published and unpublished *K13* molecular data from six GMS countries: Bangladesh, Cambodia, Thailand, Laos, Vietnam, and Myanmar. These *K13* mutation prevalence data (number of sites = 64, number of indivduals = 1832) were pooled from two sources: the NEJM TRAC clinical trial [11] and the cross-sectional survey of Tun et al. [7]. In both cases the primary sampling unit is the malaria treatment centre or health facility at each site, which has a latitude and longitude reference verified by the data contributor for accuracy. A summary of the frequency of such samples from each country is exhibited in the Additional file 1: Table S1. Some data derive from clinical trials during 2011–2014 that assessed the prevalence of mutant *K13* alleles in particular geographic locations [7, 11]. We make the assumption throughout this paper that artemisinin resistance is conferred by nonsynonymous mutations which change the primary protein sequence occurring at codons above amino acid position 440 in the *K13* gene [10, 17]. Nonsynonymous mutations are those that result in a change in amino acid in the protein which may alter its activity. This assumption may change as a more refined metric of artemisinin resistance is defined by further genetic research.

### Geospatial mapping

Nonsynonymous mutations occurring at codons above amino acid position 440 in the *K13* protein together with the geographical information system (GIS) coordinates of each sampling site, were input in a geostatistical model, yielding a predictive map on a 5 × 5 km grid of estimated mutation distribution and hence prevalence of parasite isolates assumed to be artemisinin-resistant. The model we utilize here is ‘kriging’ interpolation (giving best linear unbiased estimates at grid locations) implemented in MATLAB release 2013b and originally developed by Matheron in 1963 [18] (see Additional file 1).

An assumption of complete absence of information is made for regions beyond the boundaries of the domain in generating these maps. There is therefore smoothing of any potential ‘edge effects’ in addition to the smoothing which is typically observed with kriging [19].

The geospatial models applied by the WorldWide Antimalarial Resistance Network (WWARN) to the Southeast Asia region have utilized *K13* mutation prevalence data to produce continuous spatial maps at a 5 × 5 km resolution for estimating the prevalence of parasites carrying such genetic markers and hence assumed to be artemisinin-resistant [7]. The choice of a 5 × 5 km grid resolution (as used previously by MAP [21]) provides a sufficiently detailed overview of the Greater Mekong Subregion required for smart surveillance purposes. The underlying approach, which is applicable to mapping antimalarial drug resistance in general, has two steps:

1. Obtain data for a metric of choice to generate the map. This metric could incorporate current data on artemisinin resistance such as parasite clearance rates in patients, parasite survival rates after drug exposure, in vitro susceptibility tests, prevalence of isolates that carry a *K13*-propeller polymorphism, or any combination of these parameters. All such measures can be estimated from sampling conducted at each site to derive an estimate of resistance prevalence, defined as the proportion of total parasite isolates that are recorded as resistant.
2. Generate a continuous map over a domain of interest from collected data, using a geospatial model. The outcome is a surface that predicts the prevalence of the metric of interest across the entire geographical region. Typically these models will operate in a geostatistical framework through spatial interpolation or by ‘fitting’ an underlying model to the data obtained for the given sites. A predictive map of the spatial distribution of artemisinin resistance is then obtained as a continuous surface on a regular grid covering the region of interest. This is computationally achieved by deriving a statistical estimate of the chosen metric at each grid location. A corresponding ‘uncertainty’ map is simultaneously generated with uncertainty represented by a suitable variability statistic. This map therefore shows the uncertainty connected with the former map on a pixel-by-pixel basis (i.e., the grid resolution of the map).

The resistance map can be viewed as a ‘landscape’ with local peaks (or troughs) corresponding to those areas with highest (or lowest) estimated prevalence of artemisinin resistance.

Each resistance map (together with its associated uncertainty map) serves as a vehicle to identify specific sites or regions where further measurements could be proposed. Such ‘second phase sampling’ would aim to identify locations in the original resistance distribution where current information on prevalence is most deficient. Therefore, additional sampling at those sites could reduce the uncertainty of the resistance estimate and produce a more accurate estimate of the spatial distribution. The latter approach treats the uncertainty map as an associated landscape where peak locations would now correspond to areas of greatest uncertainty. Selection criteria constraints could also be included, for example to exclude regions where geographic remoteness, low population density, or ongoing security issues would render sampling too costly or dangerous. In all cases, the main objective would be to enhance knowledge of the current distribution of antimalarial drug resistance.

### Role of the funding source

The funders of the study had no role in study design, data collection, data analysis, data interpretation, or writing of the report.

## Results

**Fig. 1:**
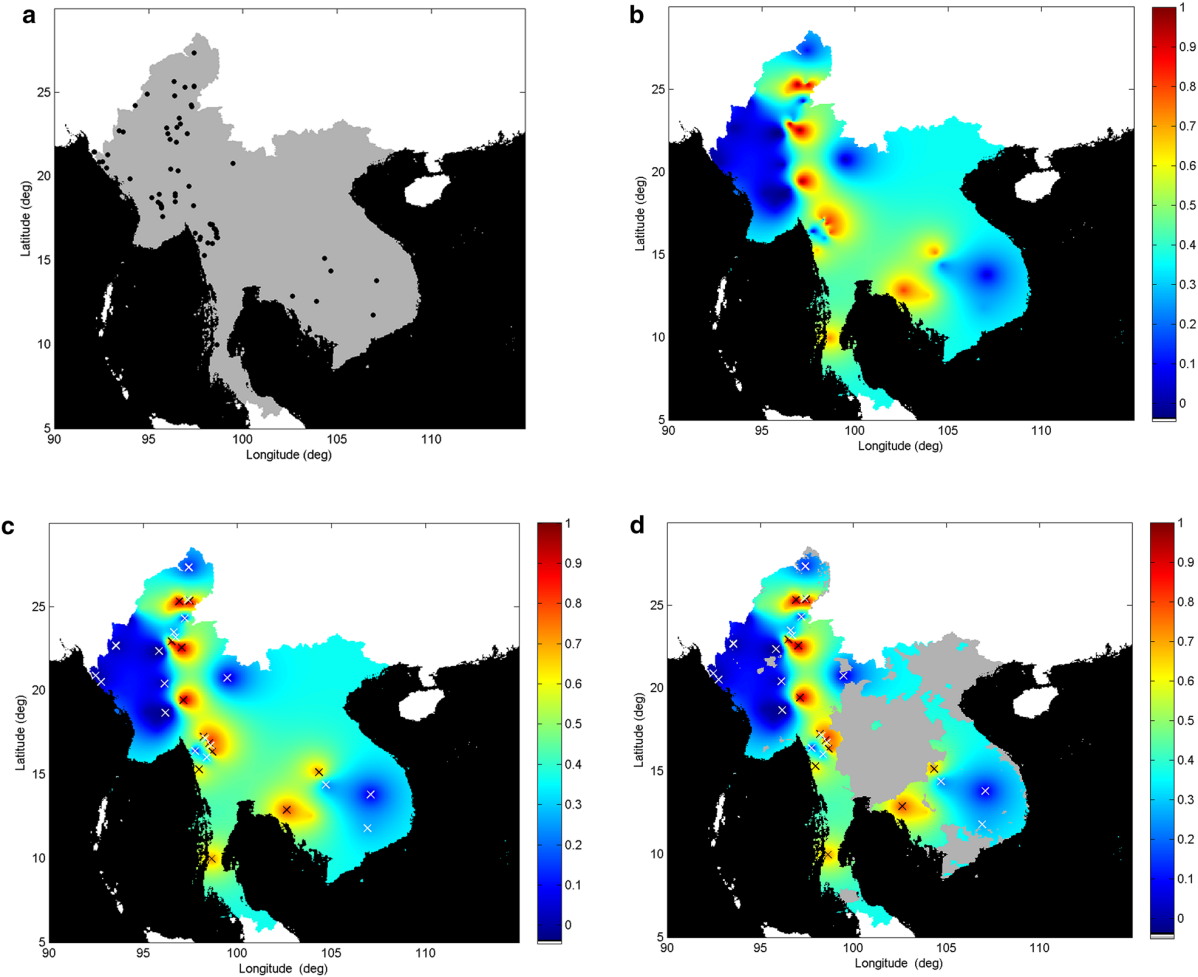
Artemisinin resistance maps (5 × 5 km grid resolution). Prevalence is displayed on the 0–1 *colour* scale. **a** The Southeast Asia domain of interest (*grey*), showing study sites (*black solid circles*). Prevalence of artemisinin resistance was determined at these sites using *K13* genetic marker data collected in 2011–2014. **b** Geospatial map generated by a kriging model of the estimated distribution of artemisinin resistance, based on the sample data in **a**. **c** The locations where artemisinin resistance is estimated to be highest (*black crosses*) and lowest (*white crosses*), based on the locations of the local maxima and local minima of the geospatial map in **b**. **d** Geospatial map of **c** with a spatial constraint imposed (*light grey*) to exclude those regions where transmission is estimated to be unstable (as defined by MAP, 2010 [21])

The strategic modelling approach is illustrated by applying smart surveillance to the spatial distribution of the prevalence of *K13*-propeller polymorphisms associated with artemisinin resistance in the GMS. Second phase sampling strategies are then motivated by knowledge of the peak and trough locations of resistance, and of those regions with greatest information deficiency. Prevalence of isolates with a nonsynonymous mutation after codon 440 (as used in Tun et al. [7]) was employed as an estimate of the prevalence of artemisinin resistance in a geospatial model with the site data, as shown in Fig. 1a.

**Fig. 2:**
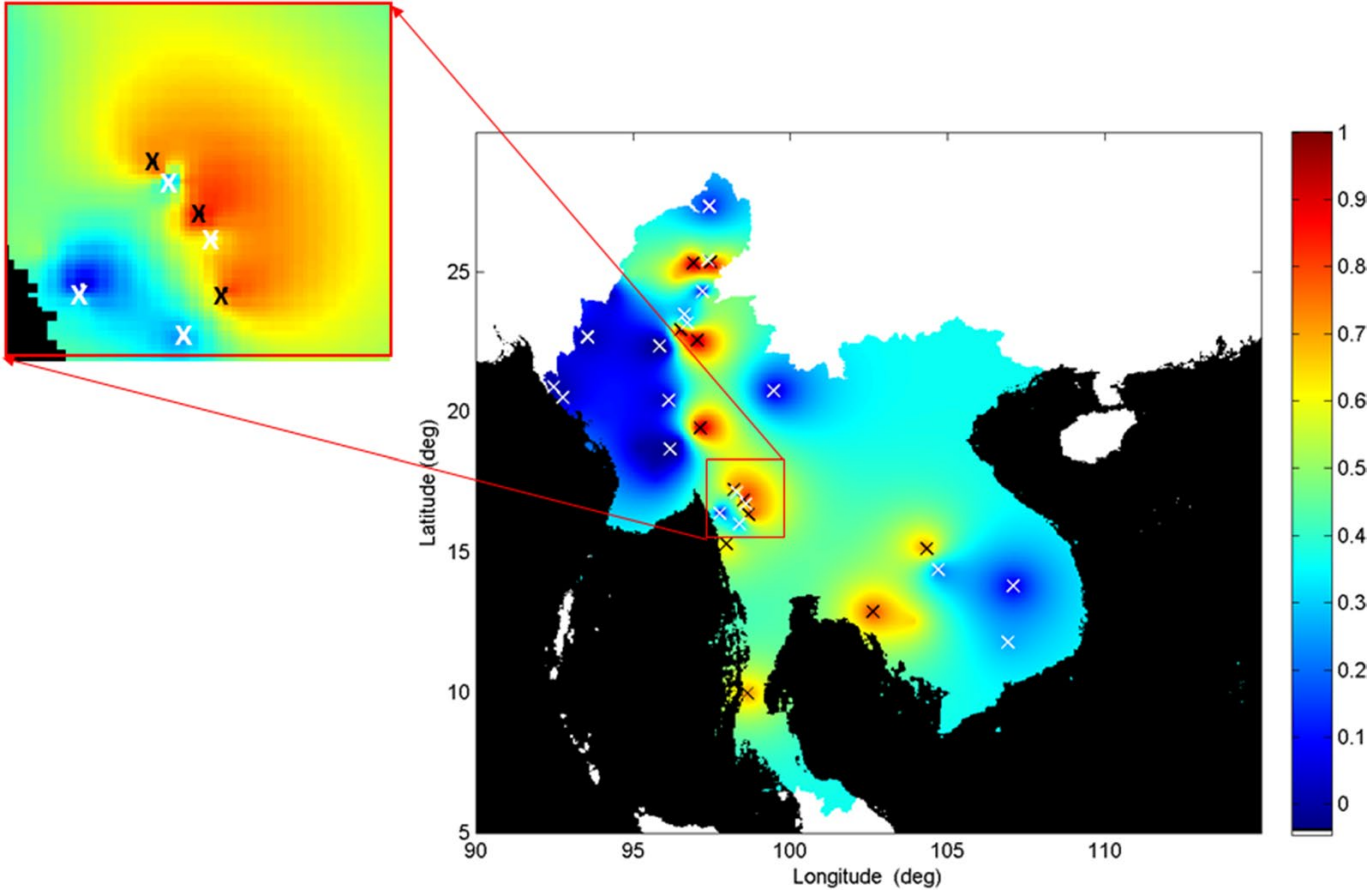
Some maxima and minima may be located close to each other in regions where spatial autocorrelation is low

The output kriging map of resistance prevalence is shown in Fig. 1b. Figures 1c and 2 (isolated in inset) show the resistance map with superimposed local maxima (12 black crosses) and minima (16 white crosses), as defined by the topology of the geospatial surface, which correspond to peak (and trough) locations with highest (and lowest) estimated prevalence of resistance, respectively. These are locations where further studies would be a priority, to confirm and update current information on estimated resistance prevalence. Knowledge of the locations of these peaks and troughs would have immediate utility in enabling the most appropriate antimalarial to be used by local clinicians. Any additional spatial information relevant to resistance prevalence could also be incorporated. For example, current knowledge of regions where malaria transmission is known to be low, as determined from other regional surveys or sources [20], could be incorporated as an additional constraint to be imposed on site identification as shown in Fig. 1d, where the spatial limits of unstable transmission suggested by the malaria atlas project (MAP) are imposed in grey [21]. Sites falling within these areas could reasonably be excluded from further studies.

**Fig. 3:**
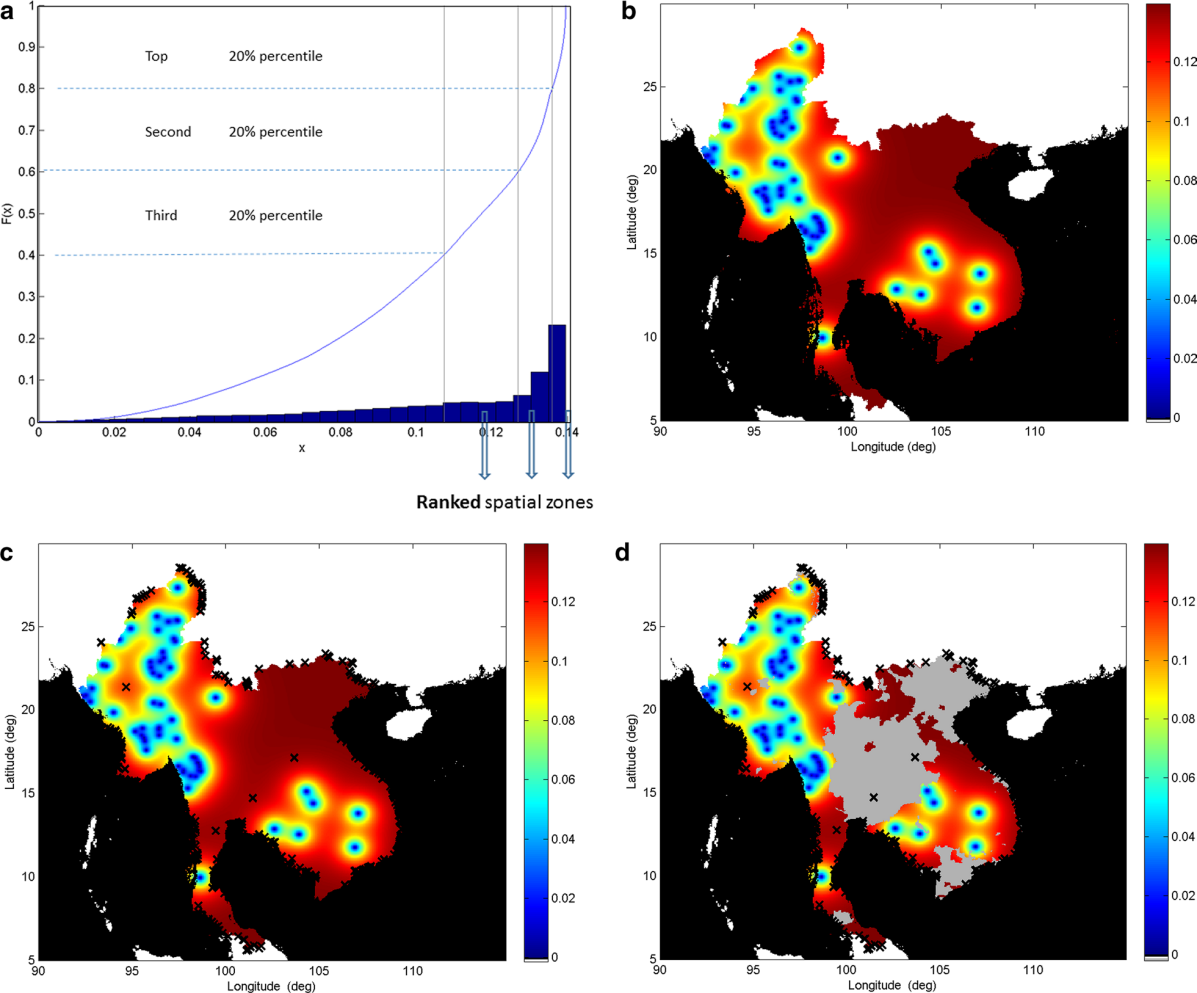
Uncertainty and uncertainty maps (5 × 5 km grid resolution). Uncertainty is represented by the kriging variance displayed on the 0–1 *colour* scale. **a** Distribution of uncertainty, represented by the kriging variance *x* associated with the uncertainty map of **a** with corresponding cumulative frequency plot *F*(*x*) (*blue line*). This key distribution is used to define uncertainty thresholds in terms of 20 % percentiles, which in turn give rise to the ranked spatial zones (shown in Fig. 4). **b** The uncertainty map corresponding to the prevalence resistance map in Fig. 1b, represented by the kriging variance. Uncertainty is lowest at the study sites where sample data were collected, as kriging is an exact interpolator. **c** Locations where uncertainty in estimated resistance is highest (*black crosses*), based on local maxima of the uncertainty map in **b**. In general, these locations are situated in the more peripheral regions of the domain that are furthest from the study sites. **d** The uncertainty map of **c** with a spatial constraint imposed (*light grey*) to exclude those regions where transmission is estimated to be unstable (as defined by MAP, 2010 [21])

Figure 3a shows the key distribution associated with the uncertainty map in Fig. 3b, as a histogram (blue bars) with corresponding cumulative distribution (blue line). Figure 3b shows the corresponding ‘uncertainty map’ associated with the resistance map in Fig. 1b, where uncertainty is represented by kriging variance and interpreted as a landscape describing spatial information deficiency. Because kriging is an exact interpolator, the uncertainty map surface has minima (troughs) at those locations where data were obtained, reflecting that uncertainty is least in these locations. However, the local maxima (Fig. 3c, black crosses) in this uncertainty map could be optimal target locations for further sampling to reduce uncertainty in the resistance map. Figure 3d shows how the sampling strategy could be modified further by excluding sites in the unstable transmission region (grey). In general, the altitude of the map surface increases in a smooth and monotonic manner as distance from sampling sites is increased, as can be seen by the deeper red colouration shown in the more peripheral regions of the GMS. This tendency, which results from a smooth uncertainty landscape, is most evident in the central northern border extremities and southern peninsular border region of the GMS, where clustering of the local maxima occurs. In Fig. 3a, the distribution can be stratified into quantiles of a fixed arbitrary percentile threshold (here 20 %), each with a corresponding zone on the uncertainty map. The most uncertain locations in the map domain are associated with the highest (top) ranked percentiles which thereby give rise to zones corresponding to ‘equivalent uncertainty’, which are displayed in the map. These uncertainty zones can be ranked and, when viewed in combination with the prevalence map, can define spatial zones as priority targets at the strategic level.

**Fig. 4:**
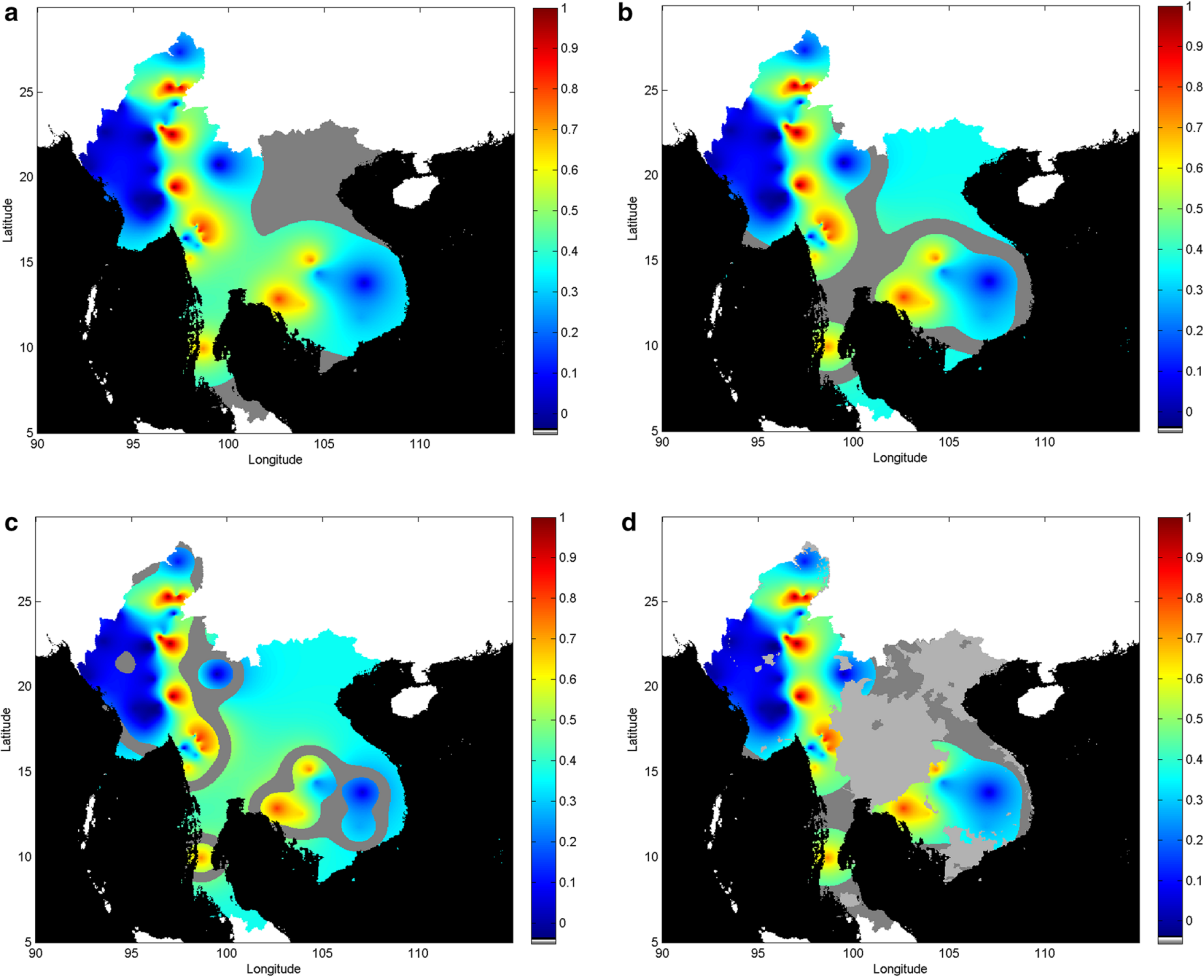
‘Equivalent uncertainty’ zones (5 × 5 km grid resolution). Prevalence is displayed on the 0–1 *colour* scale. Zones of ‘equivalent uncertainty’ are shown superimposed (*dark grey*) onto the prevalence resistance map of Fig. 1b. The three highest ranked percentiles (in 20 % bands) associated with the uncertainty map of Fig. 3a are shown. Each zone would represent a priority region to be targeted with second phase sampling aimed at improving the accuracy of the current resistance map: **a** top percentile (0–20 %), **b** second percentile (21–40 %), **c** third percentile (41–60 %). **d** Top percentile (0–20 %) with the spatial constraint imposed (*light grey*) to exclude those regions where transmission is unstable (as defined by MAP, 2010 [21])

These corresponding spatial zones are shown superimposed (dark grey) in Fig. 4, where the landscape of Fig. 1b has been zoned into regions of equivalent uncertainty based on the topography of its associated uncertainty map (Fig. 3b). The zones for the highest three percentile bands of uncertainty using a percentile band width of 20 %, range from the highest band (uppermost 20 %) in Fig. 4a, to the second highest band (21–40 %) in Fig. 4b, to the third highest band (41–60 %) in Fig. 4c. The zones typically include boundary regions of the GMS but now more widely cover internal locations. To demonstrate the utility of incorporating further spatial information or constraint into the assessment, the top band (zone previously shown in Fig. 4a) is now recalculated after superimposing the unstable transmission region defined by MAP and shown in Fig. 4d. Sampling can be made more (or less) dense in these zones, which may be deemed to be more (or less) critical because of relevant known information or constraints that may be imported into the domain or scenario under consideration. The practical application of such ‘threshold zoning’ can be decided in light of approaches currently being used by policy makers to counter drug resistance, for example, to define common or consistent approaches that the policy maker may wish to be used across regions of particular interest.

## Discussion

To facilitate policy making, there is a clear need to connect complex spatial model outputs with strategic allocation of resources, an approach that has been widely used in making strategic decisions in other areas [22] but to a lesser extent in disease risk management [23, 24]. Application of data visualization tools that provide geospatial information can inform policy to ensure optimal drug use, deployment of resources, and rapid response to newly emerging resistance. At the same time, those areas free of resistance can be engaged with recommended therapeutic, preventive, and vector control measures. Smart surveillance is a generic approach to enable health managers to develop optimal strategies to combat any disease. Dependent on the disease, this can be structured to known constraints to define the best actions which reduce geographic spread. Simultaneously, this must enable a better understanding of the disease epidemiology to be acquired. This method is a general one, and could be applied to other genetic markers correlated with resistance to ACT partner drugs. In this context, smart surveillance can provide a first step toward wider application of modelling to extend efforts in malaria control and combat antimalarial resistance. The utility of the model can be enhanced by exploring clearly defined alternatives based on thresholds that are deemed to be relevant. These thresholds must somehow incorporate the notion of risk because any such decision making process takes place under uncertainty. A natural vehicle for achieving this goal is to stratify the associated uncertainty map surface based on the distribution of its surface values (pixel values).

Several extensions could be incorporated to improve evidence provided for decision-making processes. For example, if the sequential order of site selection is important in defining a combat strategy, then optimal sites selected will be different from those in a simultaneous choice model. The iterative modelling necessary to update the resistance map and determine site selection would take into account the additional information gained as each site is identified. Decision-making could be assisted further by determining regions within the domain of interest where estimated prevalence together with associated uncertainty are to be considered in tandem. In particular, policy makers may wish to identify areas in which prevalence of resistance falls below a threshold level (e.g., 5 %) perceived as significant, but where the associated uncertainty exceeds a desired degree of confidence. Combining the prevalence and uncertainty maps into a single ‘relative uncertainty’ map, calculated as the ratio of prevalence to uncertainty, can identify these. The decision maker may wish to incorporate other covariates deemed to be relevant [25]. In such extensions, more sophisticated search algorithms will be required to identify the local maxima and minima in corresponding nonlinear objective functions of a multivariate parameter space. A possible limiting factor to spatial inference is sampling bias of data, which may arise through uneven or skewed sampling at different locations as well as pooling of data obtained across different time and spatial scales. Dependent on the spatial interpolative approach adopted however, this can still be controlled by a variety of statistical approaches. For example with kriging, the kriging variance can be elaborated so that it is ‘weighted’ to counter for such effects [25]. Alternative geostatistical models such as PYMC [26] or R-INLA [27] (or combinations of model ensembles) relying on different underlying assumptions or parameterisations could be employed to generate additional maps for comparison. In this context, information on sample sizes and geographical barriers to transmission (e.g., mountains, lakes, etc.) could be incorporated.

In order to be operationally relevant, this method will rely greatly on the capacity to collate data of interest in a timely manner. While data sharing is increasingly embraced by the scientific community [28], policy makers [29, 30], and funders [31, 32], the real-time implementation and necessary systems to securely share data still lag behind [33]. In the surveillance of antimalarial resistance, the number of organizations collecting data of interest are relatively limited to national malaria control programs, the World Health Organisation (WHO), non-governmental organisations, and research groups. Developing a mechanism to share published and unpublished data will be essential to achieve the objectives described here. In the absence of a global policy framework or operational guideline for sharing public health data, we propose to use the data sharing platform and governance structures developed by WWARN to facilitate such endeavours, and to pilot and validate this methodology in Southeast Asia. However, political support and endorsement from endemic countries and the WHO will be critically important to ensure the success of this approach.

In all cases, this methodology relies on the accuracy of the map surface generated, which in turn depends on the data available and the underlying assumptions of the geospatial model employed. An inherent limitation of the current methodology comes from its dependence on an indirect metric to represent drug resistance (here we have used prevalence of parasite isolates with any nonsynonymous *K13*-propeller mutation). The sensitivity and specificity of this definition may be enhanced by using only the subset of *K13*-propeller mutations that have been associated with artemisinin resistance in clinical and in vitro studies, or combining them with other molecular markers. This limitation can be partially mitigated by employing alternative metrics and comparing their respective maps to see how they may change or concur across any regions of common overlap. In practice, spatial data will need to be aggregated in time to provide sufficient accuracy in the maps conveyed. There is therefore a ‘trade off’ between gaining maximum utility (enabling spatial patterns to be reviewed and resources to be allocated) and defining an appropriate time scale for aggregation (enabling maps to convey sufficient information). This is to be decided in view of the given data and selected metric. In any event, the main value of smart surveillance remains unaltered, which is to utilize *any such metric* to optimally benefit the decision making process.

As with any statistical modelling approach, limitations to inference are defined by the assumptions used, and the quality, quantity, and timeliness of data. If emergence and transmission of resistance change rapidly, the dynamic situation will limit assessments of the impact of policy interventions from transmission data. Conversely, with sufficient data collected over space and time, the approach could be extended by employing a spatiotemporal model (as in [34]) to investigate how optimal sites might change over time in view of the sampling strategies and interventions performed to date.

## Conclusion

Smart surveillance provides a cost-effective and efficient approach to monitoring the geographical distribution of antimalarial drug resistance in endemic regions. The methodology can be employed to improve the quality and efficiency of drug robustness mapping and thereby assist practical operations to eliminate malaria (and more generally, any disease) in affected areas.

### Future extensions

If population genetics could be made available from samples collected, the method could also help to determine whether antimalarial drug resistance has spread or emerged independently outside the geographic domain of interest. Since these two outcomes have different underlying mechanisms, this additional information should improve ongoing malaria control and elimination efforts.

## Additional file

**Additional file 1: Table S1.** Data used in the *K13* illustrative example from (7) and (11) partitioned by individuals and sites sampled within each country.** S2.** Utility of kriging with** S3.** five step implementation procedure for smart surveillance, together with** S4.** the underlying kriging equations to be solved, also provided** S5.** in terms of variograms.** Fig. S1.** Empirical variogram of semivariance* γ*(*h*) constructed by binning all pairwise site distances in the GMS data set into 20 bins, each at a fixed increment apart, plotted against lag distance* h*.

## Abbreviations

ACT: artemisinin-based combination therapy
GMS: Greater Mekong Subregion (GMS)
*K13*: Kelch 13 gene
MAP: malaria atlas project
RSAs: ring stage survival assays
SNP: single nucleotide polymorphism
TRAC: tracking resistance to artemisinin collaboration
