# Supplementary material for: Optimal health and disease management using spatial uncertainty: a geographic characterization of emergent artemisinin-resistant Plasmodium falciparum distributions in Southeast Asia
Source: Int J Health Geogr. 2016 Oct 24;15:37. doi: 10.1186/s12942-016-0064-6 (PMC5078981; doi:10.1186/s12942-016-0064-6)
Supplement: Supplementary file 1 — Additional file 1: Table S1. Data used in the K13 illustrative example from (7) and (11) partitioned by individuals and sites sampled within each country. S2. Utility of kriging with S3. five step implementation procedure for smart surveillance, together with S4. the underlying kriging equations to be solved, also provided S5. in terms of variograms. Fig. S1. Empirical variogram of semivariance γ(h) constructed by binning all pairwise site distances in the GMS data set into 20 bins, each at a fixed increment apart, plotted against lag distance h. [file 12942_2016_64_MOESM1_ESM.docx]

**Supplementary Material**

**S1. Data** **summary**

| **Country** | **Tun et al (2015)** | | | **TRAC (Ashley et al 2014)** | | | **TOTALS** | | |
| --- | --- | --- | --- | --- | --- | --- | --- | --- | --- |
|  | *n*>440 | *n* inds | *n* sites | *n*>440 | *n* inds | *n* sites | *n*>440 | *n* inds | *n* sites |
| Bangladesh | 0 | 25 | 1 | 0 | 56 | 1 | 0 | 81 | 2 |
| Myanmar | 249 | 758 | 49 | 20 | 79 | 1 | 269 | 837 | 50 |
| Thailand | 122 | 157 | 4 | 99 | 177 | 3 | 221 | 334 | 7 |
| Cambodia | - | - | - | 183 | 460 | 4 | 183 | 460 | 4 |
| Vietnam | - | - | - | 28 | 120 | 1 | 28 | 120 | 1 |
| Totals | 371 | 940 | 54 | 330 | 892 | 10 | 701 | 1832 | 64 |

**Table S1.** Data used in the K13 illustrative example from (7) and (11) partitioned by individuals and sites sampled within each country. Key: *n>440* =number of individuals with a gene mutation present at a codon number>440, *n inds* =number of individuals tested at sites, *n sites* =number of sites.

**S2. Utility of kriging for smart surveillance**

Kriging is a widely employed statistical interpolation method which has three main forms, namely *simple* where the mean of the Gaussian spatial process is known*, ordinary* where the mean is unknown but is constant and *universal* where the mean is unknown and an underlying spatial trend also exists (19). In practice, the mean is typically unknown but can reasonably be assumed to be constant, so the most frequently applied is *ordinary* kriging. The method has the major practical benefits of being a best linear unbiased estimator, is robust with respect to the data distribution (e.g. non-normality) and has a relatively fast computation time (19). These features make it an ideal technique for implementation within smart surveillance.

In addition, kriging can be widely applied and is widely available in many mainstream software packages such as ArcGIS and is freely available in open source software such as Q-GIS or the package GSTAT in R. Such tools may also allow further enhancement of estimates, for example through enabling inbuilt transformation to rescale map outputs to a finer spatial resolution in the analysis.

**S3.**  **Five Step Smart Surveillance Implementation Procedure**

1. Obtain geo-referenced epidemiological data for a metric of choice

2. Employ a geospatial model to obtain a continuous map over the domain of interest at a convenient resolution

3. Generate the associated uncertainty map in terms of a suitable statistic (e.g. variance as a proxy for uncertainty)

4. Stratify the uncertainty map into ranked quantiles (e.g. percentiles) to identify the zones of highest uncertainty

5. Prioritize strategic policy to best target these areas and locations

**S4. Kriging Equations**

Briefly, if z(x_k_), *k* =1, 2 … *n* are the observed values of the variable z at locations x_1_, x_2_,… x_n_, we seek coefficients λ_k_ for *k* = 1,2, … *n* such that the estimate ẑ_0_ of z_0_ (=z(x_0_)) at any point x_0_

$$\hat{z}_{0}=\sum_{k=1}^{n} \lambda_{k}z(x_{k}) (1)$$

subject to the constraint (to ensure unbias)

$$1=\sum_{k=1}^{n} \lambda_{k} (2)$$

minimizes the mean squared prediction error (referred to as ‘the kriging variance’)

$${\sigma_{e}}^{2}=Var \left( z_{0-}\hat{z}_{0} \right) (3)$$

The kriging coefficients (λ_k_ for *k* = 1,2, … *n*) are found by solving the linear system of equations

$$\left[ \begin{matrix} Cov\left( z\left( x_{1} \right),z\left( x_{1} \right) \right) & \ldots& Cov\left( z\left( x_{1} \right),z\left( x_{n} \right) \right) & 1 \\ \vdots& \ddots& \vdots& \vdots\\ Cov\left( z\left( x_{n} \right),z\left( x_{1} \right) \right) & \ldots& Cov\left( z\left( x_{n} \right),z\left( x_{n} \right) \right) & 1 \\ 1 & \ldots& 1 & 0 \end{matrix} \right]\left[ \begin{aligned} \lambda_{1} \\ \vdots\\ \lambda_{n} \\ \beta\end{aligned} \right]=\left[ \begin{aligned} Cov\left( z\left( x_{1} \right),z_{0} \right) \\ \vdots\\ Cov\left( z\left( x_{n} \right),z_{0} \right) \\ 1 \end{aligned} \right] (4)$$

or

$$\boldsymbol{C\lambda}=\boldsymbol{D}(5)$$

where β is a Lagrange multiplier employed to guarantee the unbiasedness constraint of equation (2). The kriging variance is then

$${{\sigma_{e}}^{2} =\sigma}^{2}- \left( \sum_{k=1}^{n} \lambda_{k}Cov(z\left( x_{k} \right),z_{0})+\beta\right) (6)$$

**S5. Kriging equations in terms of semivariograms**

The semivariogram can be written in terms of the covariance function as

$$\gamma_{ij}=\frac{1}{2}Var\left( z\left( x_{i} \right)-z\left( x_{j} \right) \right) =1/2( \sigma^{2}+\sigma^{2}-2Cov\left( z\left( x_{i} \right)-z\left( x_{j} \right) \right)$$

$$=\sigma^{2}-Cov\left( z\left( x_{i} \right)-z\left( x_{j} \right) \right)$$

$\Rightarrow Cov\left( z\left( x_{i} \right)-z\left( x_{j} \right) \right) =\sigma^{2}-\gamma_{ij}$

Therefore, the kriging equations (4) can be rewritten equivalently in terms of the semivariogram as

$$\left[ \begin{matrix} \sigma^{2}-\gamma_{11} & \ldots& \sigma^{2}-\gamma_{1n} & 1 \\ \vdots& \ddots& \vdots& \vdots\\ \sigma^{2}-\gamma_{n1} & \ldots& \sigma^{2}-\gamma_{nn} & 1 \\ 1 & \ldots& 1 & 0 \end{matrix} \right]\left[ \begin{aligned} \lambda_{1} \\ \vdots\\ \lambda_{n} \\ \beta\end{aligned} \right] =\left[ \begin{aligned} \sigma^{2}-\gamma_{10} \\ \vdots\\ \sigma^{2}-\gamma_{n0} \\ 1 \end{aligned} \right]$$

and because $\sum_{i=1}^{n} \lambda_{i}$ =1,

⇔$\left[ \begin{matrix} -\gamma_{11} & \ldots& -\gamma_{1n} & 1 \\ \vdots& \ddots& \vdots& \vdots\\ -\gamma_{n1} & \ldots& -\gamma_{nn} & 1 \\ 1 & \ldots& 1 & 0 \end{matrix} \right]\left[ \begin{aligned} \lambda_{1} \\ \vdots\\ \lambda_{n} \\ \beta\end{aligned} \right] =\left[ \begin{aligned} -\gamma_{10} \\ \vdots\\ -\gamma_{n0} \\ 1 \end{aligned} \right]$

or more compactly

$$\boldsymbol{\Gamma\lambda}=\boldsymbol{\Gamma}_{0}$$

The kriging coefficients and the Lagrange multiplier β are found by multiplying the right hand side by the inverse matrix $\boldsymbol{\Gamma}^{\boldsymbol{-1}}$

$$\boldsymbol{\lambda}=\boldsymbol{\Gamma}^{-1}\boldsymbol{\Gamma}_{\boldsymbol{0}}$$

The kriging variance ${\sigma_{e}}^{2}$ is found by first noting that

$${\sigma_{e}}^{2} {=\sigma}^{2}+\sum_{k=1}^{n} \sum_{j=1}^{n} \lambda_{k}\lambda_{j}Cov\left( z\left( x_{k} \right),z\left( x_{j} \right) \right)- 2\left( \sum_{k=1}^{n} \lambda_{k}Cov(z\left( x_{k} \right),z_{0}) \right)$$

$${=\sigma}^{2}+\left( \sum_{i=1}^{n} \lambda_{i}Cov\left( z\left( x_{i} \right),z_{0} \right)-\beta\right)- 2\left( \sum_{k=1}^{n} \lambda_{k}Cov(z\left( x_{k} \right),z_{0}) \right)$$

$${=\sigma}^{2}- \left( \sum_{k=1}^{n} \lambda_{k}Cov(z\left( x_{k} \right),z_{0})+\beta\right)$$

${=\sigma}^{2}$- $\boldsymbol{\lambda}^{\boldsymbol{T}}$**D**

which on replacing covariance terms in vector **D** with

$\boldsymbol{D}=\left[ \begin{aligned} Cov\left( z\left( x_{1} \right),z_{0} \right) \\ \vdots\\ Cov\left( z\left( x_{n} \right),z_{0} \right) \\ 1 \end{aligned} \right]$= $\left[ \begin{aligned} \sigma^{2}-\gamma_{10} \\ \vdots\\ \sigma^{2}-\gamma_{n0} \\ 1 \end{aligned} \right]$

gives

$${\sigma_{e}}^{2} =\sigma^{2}-\left[ \begin{matrix} \lambda_{1} & \ldots& \lambda_{n} & \beta\end{matrix} \right]\left[ \begin{matrix} \sigma^{2}-\gamma_{10} \\ \vdots\\ \sigma^{2}-\gamma_{n0} \\ 1 \end{matrix} \right]$$

$$=\sigma^{2}-\sigma^{2}\sum_{i=1}^{n} \lambda_{i}+\sum_{i=1}^{n} {\lambda_{i}\gamma}_{i0}-\beta$$

$$= \sum_{i=1}^{n} {\lambda_{i}\gamma}_{i0}-\beta$$

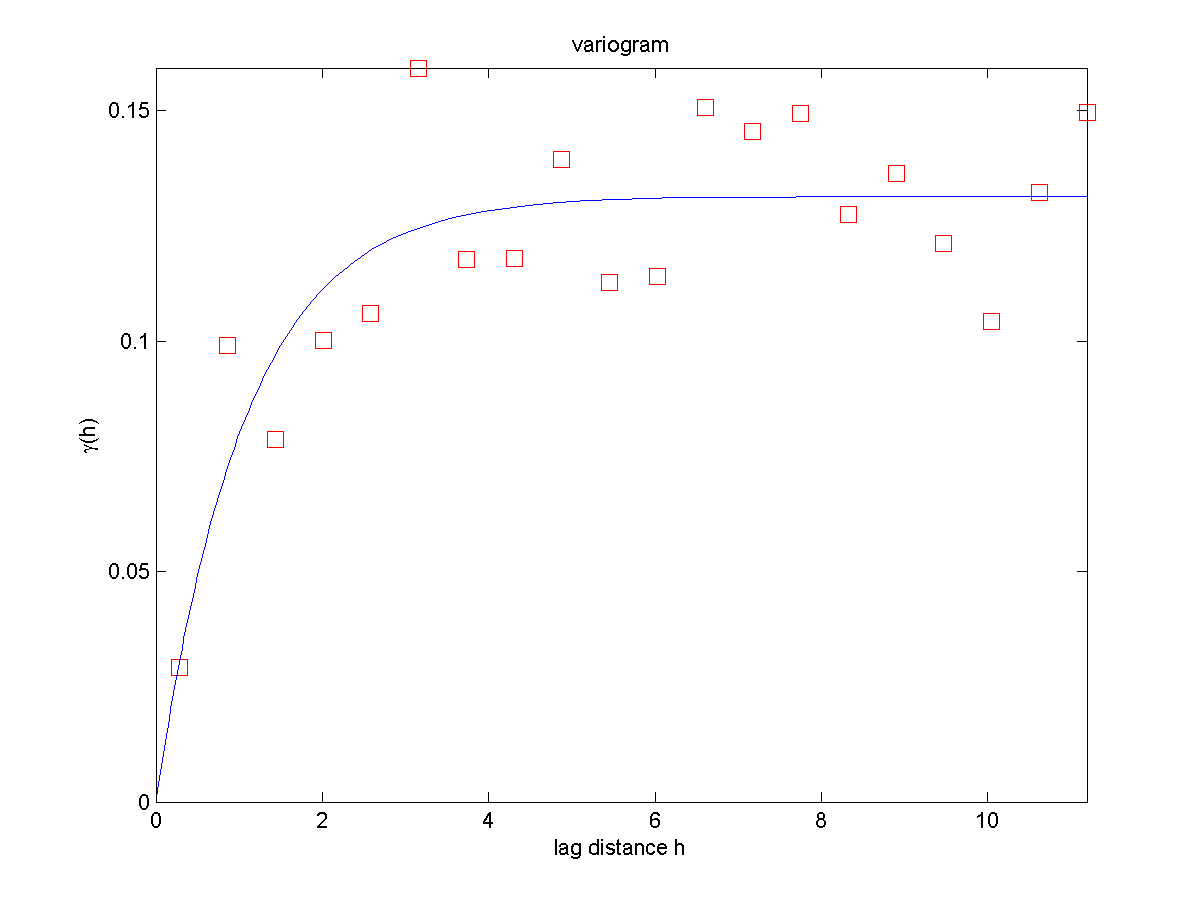


**Figure S1.** Empirical variogram of semivariance *γ(h)* constructed by binning all pairwise site distances in the GMS data set into 20 bins, each at a fixed increment apart, plotted against lag distance *h*. The plot describes how variation in estimated K13 mutation prevalence observed at different sample sites depends on (Euclidean) separation distance between those sites. The spatial dependence is modelled by the fitted exponential function (blue line)

$$\gamma(h){=c}_{0}\left[ 1-exp\left( -\frac{h}{a_{0}} \right) \right]$$

(where *h*, *c*_0_ and *a*_0_ are parameters to be estimated) and weakens with increasing distance between sample data sites until a plateau (referred to as a 'sill') is achieved after a lag distance of approximately 4 separation units =20km (on the 5km x 5km grid resolution).
